# Status of early hearing detection and intervention in South Korea: a nationwide population-based study of national infant health checkup

**DOI:** 10.1038/s41598-020-73904-5

**Published:** 2020-10-08

**Authors:** Su-Kyoung Park, Jiwon Chang, Gi Jung Im, Joong Ho Ahn, Jun Ho Lee, Kyung do Han, Jong Woo Chung, Jin-Sook Kim, Hyunsook Jang, Seung Hwan Lee

**Affiliations:** 1 Department of Otorhinolaryngology-Head and Neck Surgery, Hallym University College of Medicine, Kangnam Sacred-Heart Hospital, Seoul, Korea; 2 Department of Otolaryngology-Head and Neck Surgery, Korea University College of Medicine, Seoul, Korea; 3 Department of Otolaryngology-Head and Neck Surgery, Asan Medical Center, University of Ulsan College of Medicine, Seoul, Korea; 4 Department of Otolaryngology-Head and Neck Surgery, Seoul University College of Medicine, Seoul, Korea; 5 Department of Statistics and Actuarial Science, Soongsil University, Seoul, Korea; 6 Division of Speech Pathology and Audiology, Hallym University College of Natural Sciences, Chuncheon, Korea; 7 Department of Otolaryngology-Head and Neck Surgery, School of Medicine, Hanyang University, 222, Wangsimni-ro,Seongdong-gu, Seoul, 04763 Republic of Korea

**Keywords:** Epidemiology, Outcomes research, Paediatric research

## Abstract

The aim of this study was to evaluate the status of early hearing detection and intervention after newborn hearing screening (NHS) in South Korea. A retrospective review of Korean national health insurance service data of all infants receiving the 4-month old national infant health checkup between 2010 and 2016 from a nationwide population-based database was conducted. Based on the results of the NHS-administered hearing questionnaires as part of the national infant health checkup, individuals were classified into “pass” (1,730,615 infants) or “refer” (10,941 infants) groups. Next, an analysis was conducted of age and the frequencies of tracking audiologic tests and surgeries of the middle ear (ME) and cochlear implants (CI). Diagnostic auditory brainstem response and audiometry, and surgeries of ME and CI were significantly performed more and earlier in the refer group compared with the pass group. For infants in the pass group who were presumed to have delayed or acquired hearing loss, the time of the first audiology tests and CI surgery was significantly delayed compared to those in the refer group; the average ages for first CI were 37 and 52 months in the refer group and pass group, respectively. Therefore, for early detection of delayed-onset hearing loss, regular hearing screening programs should be considered throughout the preschool ages.

## Introduction

Early detection and intervention of hearing impairment in infants and children is associated with better outcomes in language and communication development^1–3^. In many countries, newborn hearing screening (NHS) tests are conducted on all newborns, leading to a gradual decrease in the age when deafness or hearing loss can be detected^4^. However, not all children with hearing loss can be identified using the hearing screening test in the neonatal period, and progressive, acquired or delayed-onset hearing impairment may occur as they developed^5,6^.

In November 2007, the Korean government launched a national health checkup program for infants and children to conduct screening tests for overall growth development and evaluations of hearing^7^. The national infant health checkup program is a type of population surveillance program consisting of history taking, physical examination, screening for visual acuity, and questionnaires for medical, dental, nutritional, educational, and hearing for each screening period (4, 9, 18, 30, 42, 54, and 66 months of age). Korean citizens and registered foreigners are enrolled in the National Health Insurance Service (NHIS); all medical data covered by the NHIS (e.g., national medical checkups, hospital use), are compiled into a single NHIS information database. The national official registration classifies an individual as hearing disabled when the hearing thresholds on both sides are over 60 dB HL for bilateral hearing loss or on one side is more than 80 dB HL and on the other side is over 40 dB HL for unilateral hearing loss. The national registration records for hearing disabled are also managed by the NHIS information data system^8^.

The NHS test has been covered by the NHIS since October 2018. Prior to this time, neonatal caregivers conducted NHS tests for their babies at their own expenses, and there were no national statistical records to trace screening and conforming test results and aural interventions. For this reason, the results of the NHS test before applying health insurance were indirectly known only through hearing questionnaires of national infant health checkups of 4-month old children.

The aim of this study was to analyze the status of early detection and intervention after NHS test in Korea, using the results of hearing questionnaires during the national infant health checkup (4 months of age) from 2010 to 2016.

## Methods

### Data sources

This study was conducted using nationwide data from the Korean NHIS information database^8^. The National Health Information Database of the NHIS contains comprehensive data on health spending reimbursements and national health checkups by age and provides detailed medical information on all hospitalizations and medical utilization in Korea.

### Study design and population

**Figure 1:**
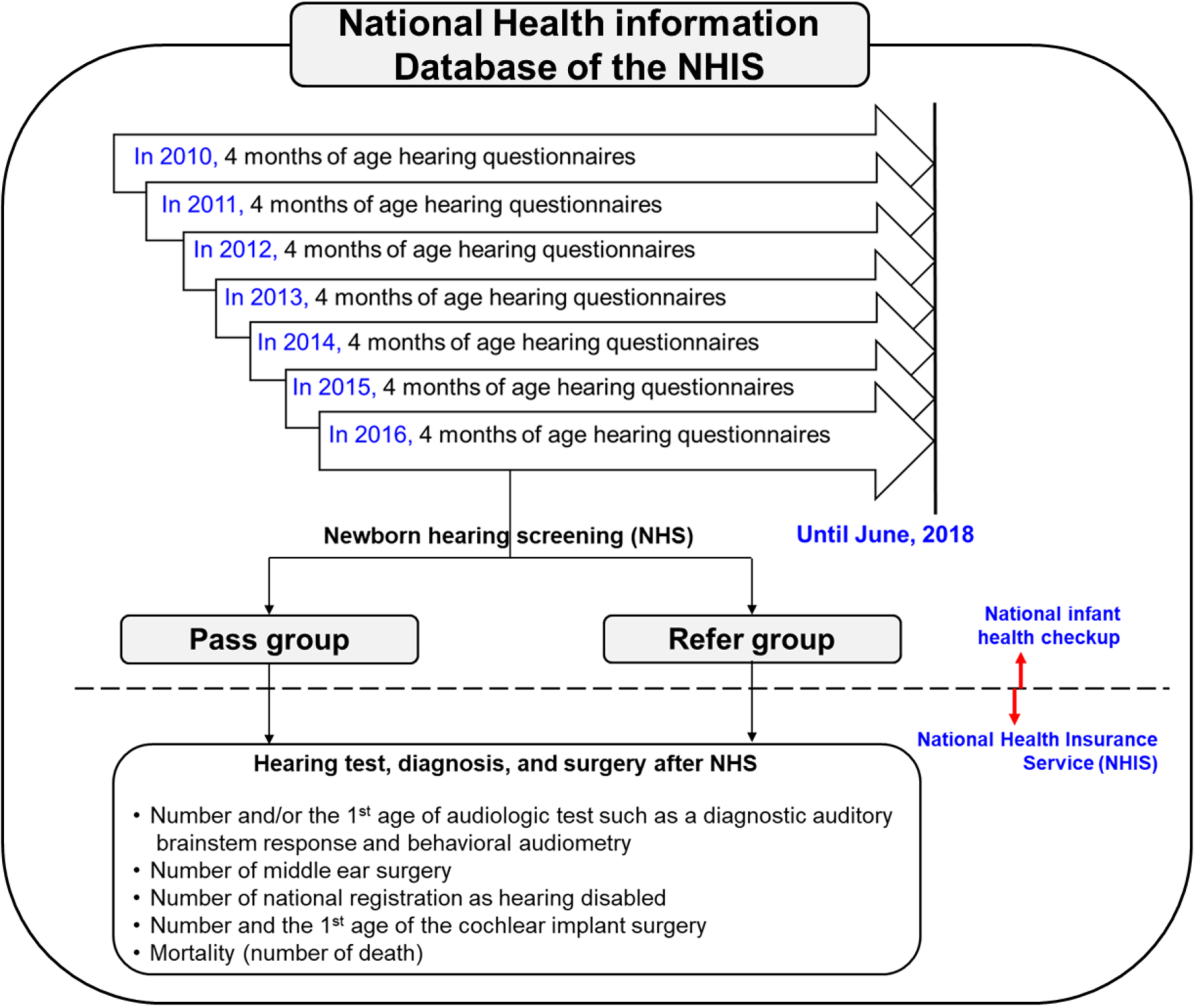
Schematic diagram of this study. According to the results of the 4-month-old NHS hearing questionnaires included in the national infant health checkup, the enrolled population was divided into two groups—pass and refer. These two groups were then analyzed for audiologic tests, middle ear surgery, CI surgery, mortality and national registration as hearing disabled based on the NHIS information database.

The population of this study included infants who underwent the 4-month age national infant health checkup between January 2010 and December 2016. First, retrospective data of the 4-month-old infant health checkup were merged with data on the health-spending reimbursements of the NHIS database from January 2010 to June 2018 (Fig. 1). The research analysis periods vary depending on the year of the infant health checkup. For example, for an infant who underwent a 4-month infant health checkup in 2010, the average medical use over 9 years was evaluated compared with an infant undergoing a 4-month infant health checkup in 2016, for whom approximately 2 years of medical records were evaluated.

**Figure 2:**
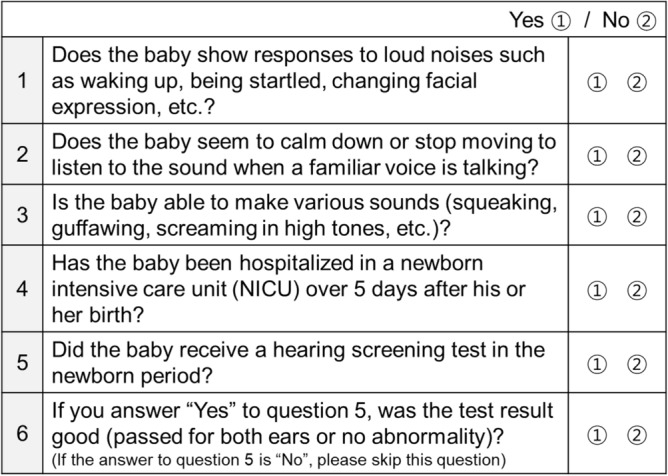
Hearing questionnaires of the 1st national infant health checkup at 4 months of age. In the hearing questionnaires of the national infant health checkup for 4-month-old infants, the fifth and sixth questions ask whether or not an NHS was conducted and what the results were, respectively.

Additionally, hearing questionnaires of the national infant health checkup for infants at 4 months of age contained questions about whether an NHS test was conducted and the results of the hearing screening test (Fig. 2). According to the results of the NHS collected from these questionnaires, the population was divided into two groups, either “pass” or “refer” (Fig. 1). Finally, for the primary analysis, the results of early hearing detection and intervention (EHDI) of the pass and refer groups after the NHS test were evaluated.

### Main outcomes and measurement

The numbers of auditory brainstem response (ABR) tests, behavioral audiometry (visual reinforcement or play audiometry, pure tone audiometry, and speech audiometry depending on the infant or child’s developmental level), national official registration as hearing disabled, and middle ear (ME) and cochlear implant (CI) surgeries as factors for hearing detection and intervention were analyzed. Mortality as an analytical factor was added because it is one of the most accurate factors in the NHIS database. Age at the first ABR test and CI surgery were also analyzed as potential factors for early detection and intervention.

Based on the national surgical code classification, ME surgeries were divided into two types: (1) otitis media with effusion, such as ventilation tube (V-tube) insertion and myringotomy, and (2) ME surgeries that have conductive hearing loss, such as obstruction of the external auditory canal, exploratory tympanotomy, tympanoplasty, myringoplasty, patch graft for tympanic membrane perforation under microscopy, mastoidectomy, stapes surgery, ossicular reconstruction, and ME tumors.

### Statistics

Statistical analysis was performed using SAS software version 9.3 (SAS Institute, Cary, NC: www.sas.com/en_us/home.html). Group comparisons for qualitative data (proportions for one variable to another variable) were performed with Pearson’s chi-square test, whereas for quantitative data such as the means of two groups, we used Student’s *t*-test or the Wilcoxon test. *P* values < 0.05 were considered statistically significant.

### Ethics approval

This study was performed by the committee of the Korean Society of Otorhinolaryngology-Head and Neck Surgery and reviewed and confirmed by the Korean Audiological Society. As a representative hospital, the study protocol was approved by the institutional review board of Hallym University Kangnam Sacred Heart Hospital (IRB file no. HKS 2016-08-105), and the need for informed consent was waived by the Institutional Review Board because of the retrospective database nature of the study. All methods were performed in accordance with the relevant guidelines and regulations.

## Results

### Status of following audiologic tests and the age of the 1st ABR

A review of the data from 2010 to 2016 led to the inclusion of 1,741,556 infants in this study, with 1,730,615 in the pass group and 10,941 in the refer group. The mean referral rate was 0.63% (Table 1). Excluding the number of duplicates, there were 14,306 infants (0.83%) in the pass group and 3443 (31.47%) in the refer group for the diagnostic ABR tests, and significantly more diagnostic ABR tests were performed in the refer group after the NHS test compared with the pass group (*P* < 0.0001, Table 1). The rate of ABR tests in the refer group was 28.90% in 2010 and it increased gradually to 36.59% in 2013 and 35.60% in 2014; however, the ABR performing rates of the pass group remained approximately 1% (Table 2). The number of infants who underwent behavioral audiometry depending on the infant’s developmental level in the refer group (579 infants, 5.29%) was significantly higher than that in the pass group (6045 infants, 0.35%) (*P* < 0.0001, Table 1).

**Table 1:** Early hearing detection and intervention status in the pass and refer groups of newborn hearing screening from 2010 to 2016.

Variables	Pass group	Refer group	*P* value
Population infants, n (% of total)	1,730,615 (99.37)	10,941 (0.63)	< 0.0001
**Audiologic test**			
Infants performed ABR, n (%)	14,306 (0.83)	3,443 (31.47)	< 0.0001
Median age at the 1st ABR, month (range)	9 (3–74)	7 (3–12)	< 0.0001
Infants underwent audiometry, n (%)	6045 (0.35)	579 (5.29)	< 0.0001
**Middle ear (ME) surgery**			
Children performed V-tube insertion or myringotomy, n (%)	9945 (0.57)	398 (3.64)	< 0.0001
Children performed ME operation except V-tube insertion and myringotomy, n (%)	11,188 (0.65)	436 (3.99)	< 0.0001
Children registered as an official hearing disabled^b^, n (%)	97 (0.01)	166 (1.52)	< 0.0001
**Cochlear implant (CI) surgery**			
Children performed CI surgery, n (%)	157 (0.01)	240 (2.19)	< 0.0001
Median age at the 1st CI surgery, month (range)	52 (32–127)	37 (27–75)	< 0.0001
Mortality, n (%)	607 (0.04)	18 (0.16)	< 0.0001

Statistical significance was assessed by Pearson chi-square or Wilcoxon-test.

*ABR* auditory brainstem response, *V-tube insertion* ventilation-tube insertion.

^a^Behavioral audiometry such as visual reinforcement, play audiometry, pure tone audiometry, and speech audiometry depending on the infant or child’s developmental level.

^b^The national official registration classifies an individual as hearing disabled when the hearing thresholds on both sides are over 60 dB HL for bilateral hearing loss or on one side is more than 80 dB HL and on the other side is over 40 dB HL for unilateral hearing loss.

**Table 2:** Status of diagnostic auditory brainstem response test after newborn hearing screening for 7 years in Korea.

Year	N, infants performed ABR/total infants (ABR performing rate of each group, %)			Median age at the 1st ABR, month (range)		
	Pass group	Refer group	*P* value	Pass group	Refer group	*P* value
2010	1590/181,872 (0.87)	322/1114 (28.90)	< 0.0001	13 (4–74)	6 (3–12)	< 0.0001
2011	1924/217,714 (0.88)	388/1202 (32.28)	< 0.0001	13 (4–72)	7 (3–11)	< 0.0001
2012	2318/231,914 (1.0)	390/1237 (31.53)	< 0.0001	10 (4–66)	6 (3–11)	< 0.0001
2013	2,588/261,345 (0.99)	517/1413 (36.59)	< 0.0001	9 (4–59)	6 (4–11)	< 0.0001
2014	2305/269,756 (0.85)	524 /1472 (35.60)	< 0.0001	9 (4–42)	6 (4–10)	< 0.0001
2015	1851/283,392 (0.65)	635/2091 (30.37)	< 0.0001	8 (3–18)	7 (4–12)	0.0311
2016	1730/ 284,622 (0.61)	667/2412 (27.65)	< 0.0001	8 (4–11)	6 (3–10)	< 0.0001

*ABR* auditory brainstem response.

Statistical significance was assessed by Pearson chi-square or the Wilcoxon-test.

In all hearing checkup periods, the median age of the 1st ABR test in the refer group was significantly earlier than that in the pass group (*P* < 0.0001, Table 1). In 2010, the median age at the 1st ABR test in the refer group was 6 months old (range 3–12); the median age in the pass group was 13 months old (range 4–74) (Table 2). In subsequent years, the median age of the 1st ABR test in the refer group remained similar, while the median age of the 1st ABR test in the pass group was getting younger, and the median age between the two groups was similar in 2015 and 2016 (Table 2).

### ME surgery due to otitis media with effusion

V-tube insertion or myringotomy due to otitis media with effusion was performed significantly more commonly in the refer group (398 infants, 3.64%) compared with the pass group (9945 infants (0.57%) (*P* < 0.0001, Table 1). The rate of ME surgeries for otitis media with effusion in the pass group was 1.33% in 2010 but it decreased gradually to 0.06% in 2016, while those of the refer group were approximately 5% between 2010 and 2014, with a decrease to 2.82% in 2015 and 1.12% in 2016 (Table 3).

**Table 3:** Status of ventilation tube insertion or myringotomy after newborn hearing screening for 7 years in Korea.

Year	N, infants underwent surgery/total infants (rate of each group, %)			Rate of infants who performed operation more than once (%)	
	Pass group	Refer group	*P* value	Pass group	Refer group
2010	2418/181,872 (1.33)	50/1114 (4.49)	< 0.0001	0.57	3.77
2011	2479/217,714 (1.14)	60/1202 (4.99)	< 0.0001	0.55	6.99
2012	1884/231,914 (0.81)	63/1237 (5.09)	< 0.0001	0.30	2.91
2013	1475/261,345 (0.56)	70/1413 (4.95)	< 0.0001	0.18	3.75
2014	947/269,756 (0.35)	69/1472 (4.69)	< 0.0001	0.08	2.17
2015	585/283,392 (0.21)	59/2091 (2.82)	< 0.0001	0.03	0.72
2016	157/284,622 (0.06)	27/2412 (1.12)	< 0.0001	0.01	0.25

Statistical significance was assessed by Pearson chi-square test.

The percentage of infants who underwent ME operations due to otitis media effusion more than once in the refer group ranged from 0.25 to 6.99%, which was much higher than that in the pass group (0.01–0.57%) (Table 3).

### ME surgery for treatable conductive hearing loss

Throughout the study, the number of infants who underwent ME surgery excluding V-tube insertion and myringotomy was higher in the refer group (436/10,941 infants, 3.99%) than in the pass group (11,188/1,730,615 infants, 0.65%) (*P* < 0.0001) (Tables 1, 4). The proportion of ME surgeries due to ME disease except otitis media with effusion in the refer group was nearly 5% except for 2015 and 2016, while that of the pass group gradually decreased from 1.44 to 0.06%. From 2010 to 2016, the percentage of infants treated with more than one surgery decreased steadily in both groups (Table 4).

**Table 4:** Status of middle ear surgery except ventilation tube insertion and myringotomy after newborn hearing screening for 7 years in Korea.

Year	N, infants underwent surgery/total infants (Rate of each group, %)			Rate of infants who performed operation more than once (%)	
	Pass group	Refer group	*P* value	Pass group	Refer group
2010	2619/181,872 (1.44)	61/1114 (5.48)	< 0.0001	0.70	6.55
2011	2727/217,714 (1.25)	70/1202 (5.82)	< 0.0001	0.66	8.40
2012	2145/231,914 (0.92)	70/1237 (5.66)	< 0.0001	0.41	3.88
2013	1725/261,345 (0.66)	75/1413 (5.31)	< 0.0001	0.28	4.95
2014	1147/269,756 (0.43)	73 /1472 (4.96)	< 0.0001	0.14	2.65
2015	664/283,392 (0.23)	60/2091 (2.87)	< 0.0001	0.05	0.86
2016	161/284,622 (0.06)	27/2412 (1.12)	< 0.0001	0.01	0.25

Statistical significance was assessed by Pearson chi-square test.

### Status of CI surgery and age of the 1st CI surgery

The total number of infants who underwent CI surgery was 157 (0.01%) in the pass group and 240 (2.19%) in the refer group. The refer group received significantly more CI surgeries than the pass group (*P* < 0.0001, Tables 1, 5). The proportion of CI surgeries in the refer group was approximately 3%, while that of the pass group was approximately 0.01% or 0.02% except in 2015 and 2016 (Table 5).

**Table 5:** Status of cochlear implant (CI) surgery after newborn hearing screening for 7 years in Korea.

Year	N, infants performed CI/total infants (CI surgery rate of each group, %)			Median age at the 1st CI, month (range)		
	Pass group	Refer group	*P* value	Pass group	Refer group	*P* value
2010	30/181,872 (0.016)	33/1114 (2.962)	< 0.0001	94.5 (52–127)	39.0 (34–59)	0.0003
2011	34/217,714 (0.016)	38/1202 (3.161)	< 0.0001	60.5 (40–125	48.5 (33–75)	0.0490
2012	33/231,914 (0.014)	34/1237 (2.749)	< 0.0001	65.0 (43–77)	40.0 (33–62)	0.0054
2013	24/261,345 (0.009)	40/1413 (2.831)	< 0.0001	52.5 (33–74)	38.0 (32–66)	0.2326
2014	23/269,756 (0.009)	49 /1472 (3.329)	< 0.0001	43.0 (33–58)	35.5 (31–41)	0.0906
2015	8/283,392 (0.003)	41/2091 (1.961)	< 0.0001	34.0 (32–44)	33.0 (4–12)	0.6147
2016	5/284,622 (0.002)	5/2412 (0.207)	< 0.0001	34.0 (33–35)	33.0 (27–35)	1.0000

Statistical significance was assessed by Pearson chi-square or Wilcoxon-test.

From 2010 to 2012, the median age of the 1st CI surgery in the refer group was significantly earlier than that in the pass group (*P* < 0.05). Between 2013 and 2016, the median age of the 1st CI surgery in the refer group was younger than that in the pass group; however, this difference was not statistically significant. Analysis of data from the national health checkup program in subsequent years revealed that the median age of the 1st CI surgery was getting younger in both groups, and the median age of the two groups in 2015 and 2016 were similar (Table 5).

### Status of the national official registration as hearing disabled and the mortality rate

The number of infants and children officially registered as hearing disabled was significantly higher in the refer group (166 infants, 1.52%) than in the pass group (97 infants, 0.01%) (*P* < 0.0001, Table 1).

The mortality rate of the refer group (18/10,941, 0.16%) was significantly higher than that of the pass group (607/1,730,615, 0.04%) (*P* < 0.0001, Table 1).

## Discussion

This study is the first official big-data analysis on the status of EHDI in infants and children after NHS in South Korea using the national health information database of the NHIS. The NHS test has been covered by the NHIS since October 2018. Prior to that time, official NHS results could not be traced in the NHIS database. Herein, we analyzed the status of the EHDI after NHS testing by linking national infant hearing checkup data with data from the NHIS^8^.

Enrolled infants who underwent the NHS test accounted for 55.4% of the Korean total live births (3,141,384) from 2010 to 2016. In this study, the diagnostic ABR-conducting rate of infants who did not pass the NHS test was 31.47%. In this study, the extraction period between 2015 and 2016 was shorter than the other periods (approximately 2–3 years in length); therefore, there were fewer infants and tests when compared with other years.

Although the NHS is important, it is more important to establish a tracking system for timely EHDI and to undertake regular surveillance to detect infants with delayed-onset or progressive hearing impairment^2,9^. JCIH recommended that the EHDI program aim to obtain follow-up diagnostic ABR tests in more than 95% of infants who do not pass the NHS test^2,9^. Therefore, the ABR-conducting rate in Korea was very low compared to JCIH’s recommendation. According to the hearing screening and follow-up survey in the United States administered between 2006 and 2012, referral rates decreased from 2.3 to 1.6%, and the diagnosis rates of referred infants increased from 4.8 to 10.3%^10^. In the early 2000s, the diagnosis rate of infants who did not pass the NHS test in the United States was also very low.

In this study, potential factors impacting the low diagnostic ABR rate were identified, and they included parents who may (1) not have enough guidance and awareness of the EHDI program or (2) not perform it due to cost because at that time, there was no national support. It is difficult to perform successful EHDI in accordance with the guidelines recommended by the parents themselves without the help of the government or related agencies. Therefore, it is necessary for the national web registration system that interfaces with the birth registration system to guide and track NHS data so that providers and parents can (1) manage infants for timely hearing screening, (2) follow-up for diagnosis and (3) identify those who missed NHS or ABR testing to improve coverage.

In the refer group, (1) the ME surgery rate due to otitis media with effusion was 3.64%, (2) the rate for all ME surgeries except V-tube insertion and myringotomy was 3.99%, (3) the rate of registration as hearing disabled in the national registration (≥ 60 dB HL in both ears) was 1.52% and (4) the percentage of infants who underwent CI surgery (at least ≥ 70 dB HL in both ears) was 2.19%. In later years, the ME surgery rate due to otitis media with effusion was approximately 5% in the refer group; however, this rate decreased in 2015 and 2016. The ME surgery rate due to otitis media with effusion in the pass group gradually decreased from 1.33 to 0.06%; it is thought that a gradual increase in the use of protein-conjugated pneumococcus vaccination was at least partially responsible for this decrease^11,12^. In infants with sensorineural hearing loss, early detection of otitis media with effusion is critical since otitis media-mediated hearing loss has a greater effect on auditory cues necessary for language development than normal middle ear or cochlear function^13,14^. In a retrospective study, otitis media with effusion was identified in 64.5% of infants who did not pass the NHS; otitis media with effusion required V-tube insertion in 22.4% of cases, and 11% of all referred infants had sensorineural hearing loss after resolution of the effusion^15^. Therefore, for infants referred from NHS with otitis media with effusion, clinicians should conduct proper follow-up to ensure that their hearing is normal when the otitis media subsides^16,17^.

The rates of all ME surgeries except V-tube insertion and myringotomy were 3.99% and 0.65% in the refer group and pass group, respectively. In the 8-year NHS results in Miyazaki prefecture, Japan, among 169 infants with hearing loss, 10 (12.5%) had bilateral conductive hearing loss and 31 (34.8%) had unilateral hearing loss; four of those with bilateral conductive hearing loss had ossicular reconstruction surgery, two of whom were no longer using hearing aids^18^. In the NHS results in Hungary, 17 out of 37 (45.9%) infants with hearing impairment had sensorineural hearing loss, 2 (5.4%) had conductive hearing loss, and 18 (48.6%) reported middle ear effusion, which improved spontaneously^19^. Hearing loss in infants and children who had ME surgery is likely to resolve after ME surgery. Therefore, early detection of children with conductive hearing loss is also important.

In this study, 11,188 infants (0.65%) in the pass group also had conductive hearing loss and underwent ME surgery. In these cases, their hearing was good enough to pass the NHS test in the neonatal period, and it could be considered that they experienced delayed-onset hearing loss or post-testing progression. Some countries recommend regular hearing screenings and checks of middle-ear status at 9, 18, 30 or 36 months of age and 60 months of age to detect delayed or progressive hearing loss early, combined with other developmental surveillance^2,18,20^.

The percentage of infants treated with CI surgery (at least ≥ 70 dB HL in both ears more than 2 years old) was 2.19% (240 infants) in the refer group and 0.01% (157 infants) in the pass group. However, among infants in the pass group who were presumed to have delayed or acquired hearing loss, the mean age of the first diagnostic ABR tests and CI surgery was significantly delayed compared to the refer group; the average age of the first CI was 37 and 52 months in the refer group and pass group, respectively (*P* < 0.0001). Fortunately, in the later years analyzed in this study, the age of first diagnostic ABR and CI surgery between the two groups became similar, a change that could be the result of efforts of the Otorhinolaryngology Society and the government—specifically, education and promotion through NHS guidelines, persistent workshops and online training. Even if infants passed the NHS test, they should receive regular hearing screenings through preschool ages for early detection of delayed-onset hearing loss.

In this study, the mortality rate of the refer group was 0.16% (18/10,941 infants), which was significantly higher than that of the pass group (0.04%, 607/1,730,615 infants). To the author’s knowledge, no reports in the literature have demonstrated that children with hearing loss under the age of 8 have a higher mortality rate than children with normal hearing. According to a report from the National Health Interview Survey in the USA, deafness in adults was associated with a higher mortality rate compared with nondeaf adults; however, this was the case for adults with postlingual deafness, not childhood deafness^21^. In another study of children aged 1–18 years in South Carolina in the United States, it was reported that the rate of injury treatment in children with hearing loss was more than double that of children with normal hearing^22^. It was reported that approximately 20–40% of children who are deaf or hard of hearing have other accompanying disabilities, and the results of their language development are not as good as those without other disabilities^23^. In a survey of 224 individuals with intellectual disability, 12.5% had hearing loss, 17% had a deterioration in vision, and 87.5% of those who were deaf-blind had profound intellectual disability^24^. Infants with a family history of sudden death or syncope may require heart screening in case they have a long QT syndrome with hearing loss, such as Jervell Lange-Nielsen Syndrome^25–27^. In addition, in a study of children who are deaf or hard of hearing in the United States, it was reported that children with profound hearing loss had significantly more cases of autism disorder than those with mild hearing loss^28^. Another study of the large linked datasets about autism spectrum and death in Australia reported that the population with autism spectrum had a 2.06 times higher mortality rate than the general population^29^. However, there was no study that could explain the high mortality rate of the refer group in this study. Therefore, further studies are needed to determine why the mortality of the refer group is so high, whether the infants who have died are hard of hearing, and what the associated factors are.

Importantly, our study has some limitations. First, the tracking period was not the same for each year of the national checkup of the infant populations included in the analysis. For example, an analysis of the medical use of infants with testing performed in 2010 included 8 years of data, while infants tested in 2016 yielded only 2 years of medical use data for analysis. As a result, the number of infants and children who underwent the ABR test, ME and CI surgery gradually decreased as the national checkup age was delayed. Therefore, in future research, a long-term analytical study is needed so that the same period can be tracked and analyzed for each checkup year.

The second limitation is that this study cannot explain why the mortality of the refer group is higher than that of the pass group, since only the results of the national infant checkup and the mortality rate itself were analyzed. These results do not indicate that hearing loss is a direct cause of high mortality, and further studies to analyze associated factors (e.g., comorbidity, history of intensive care unit admission, other surgeries) are warranted.

Nevertheless, the significance of this study is that it can be of practical help when the government establishes a policy for deaf or hard-of-hearing children by providing data on how many confirmation tests were performed and when and how much aural rehabilitation or surgeries were received according to the NHS results. Previously, it was predicted that the refer group will be diagnosed with hearing loss earlier and receive more timely aural intervention compared with the pass group; however, few large-scale studies have been conducted to characterize the types and timing of the related surgeries (e.g., ME, CI) in infants.

In conclusion, audiologic tests and ME and CI surgeries were significantly more common in the refer group than in the pass group. In infants in the pass group who had delayed-onset hearing loss, the time of the first audiologic tests and CI surgery were significantly delayed compared to those in the refer group. Therefore, for early detection of delayed-onset hearing loss, regular hearing screening programs after the NHS tests should be considered through the preschool ages.
